# The effect of contact sport expertise on postural control

**DOI:** 10.1371/journal.pone.0212334

**Published:** 2019-02-14

**Authors:** Ying Liang, Michael Hiley, Kazuyuki Kanosue

**Affiliations:** 1 Department of Physical Education, Anhui Normal University, Wuhu, China; 2 School of Sport, Exercise and Health Sciences, Loughborough University, Loughborough, Leicestershire, United Kingdom; 3 Faculty of Sports Sciences, Waseda University, Tokorozawa, Saitama, Japan; University of Minnesota, UNITED STATES

## Abstract

It has been demonstrated that expertise in sport influences standing balance ability. However, little is known concerning how physical contact in sport affects balance ability. The aim of this study was to examine whether differences between contact and limited-contact sport experiences results in differences in postural control. Twenty male collegiate athletes (10 soccer/contact, 10 baseball/limited contact) and ten male untrained students stood quietly on a force plate under various bipedal and unipedal conditions, with and without vision. Significant differences for sway area and COP speed were found between the soccer players and the other two groups for unipedal stances without vision. Soccer players were found to have superior postural control compared with participants involved in limited contact sport or no sport at all. Contact sports may lead to increased postural control through enhanced use of proprioceptive and vestibular information.

## Introduction

Expertise in sports which required good balance, e.g. gymnastics and dance, is of particular benefit to postural control [1,2,3,4,5,6]. Posture is controlled by integrating visual, proprioception and vestibular information [7]. These three types of information are obtained from the environment and the task [1,3,8,9,10,11]. In light of this fact, it should be expected that if non-gymnasts and non-dancers spend a great deal of time in environments with continual external disturbance, they may also develop greater adaptive ability in postural control. However, one limitation in postural control research is that previous studies may have been “contact sport biased” in non-gymnastics sports such as soccer, handball or American football [1,2,3]. Balance studies have been conducted on a variety of sports [12,13], however, the amount of physical contact involved has not been taken into account when attempting to clarify how expertise in sport contributes to postural control. Ideally, to obtain this information, a prospective study of contact experience is necessary. An extensive review of the literature on balance and different sports has been conducted [14], however, the consistency of measures (equipment and task difficulty) and sports of different amounts of contact has not been conducted. In the review of balance and various sports the consideration of the amount of contact involved in a sport is not addressed [14].

“Contact sport” is a term used in both competitive activity and in medical terminology to indicate a sport that emphasizes or requires physical contact between players [15]. Different classification has been used in different situations in relation to contact in sports. In order to categorize the degree of contact in different sports optimally, the system adopted by the United States for medical terminology has been used. This system uses the term “contact sport” to refer to sports such as soccer and basketball, in which athletes routinely make contact with each other or inanimate objects, but usually with less force than in “collision sports”, such as rugby and American football. The term “limited-contact sport” denotes sports such as squash and baseball, in which contact with other athletes is infrequent or inadvertent [15]. The focus of the present study was to make comparisons between soccer players, baseball players, and controls (novices) to establish whether differences between contact and limited-contact sport experiences result in differences in postural control.

Postural responses induced by external perturbations have been thoroughly investigated in relation to standing positions such as bipedal and unipedal stances [1,2,5,16,17,18,19], which have aimed to differentiate the complexity of postural performance in line with decreases in the “supporting area”. However, in many control studies, postural sway in bipedal stance showed no difference among athletes of different sports or compared with novices, while uni-pedal stance has been shown to be a less stressful task for gymnasts compared to non-gymnasts or for high-level soccer players compared to low-level soccer players [1,2,10]. Garcia et al. [6] reported that gymnastics training benefits postural control of bipedal standing only in younger children and suggested that more challenging stances should be investigated. Similarly, bipedal and unipedal tests may not be sufficiently challenging to compare the postural differences that may be present in the contact and limited-contact sports. With this in mind, the use of toe-stance [20] (i.e. standing on toes), which is more challenging than unipedal and bipedal stances, may be helpful to further determine to what extent expertise in sport contributes to postural regulation.

It is suggested that the cerebellar-cortical loop is responsible for adapting postural responses based on prior experiences [21]. The effect of sport experiences on postural control will relate to how the athlete more effectively uses sensory information. For example, the somatosensory inputs involved in the perception of the support conditions may play an important role in postural control in athletes participating in contact and limited-contact sports. Furthermore, since visual input is extremely important feedback information, postural control always deteriorates in eyes-closed conditions compared with eyes-open conditions [7,22,23,24]. Thus, the aim of this study was to investigate how postural performance differs from the amount of contact in sport (soccer and baseball) across bipedal, unipedal, unipedal on foam and toe stances with both eyes-open and eyes-closed.

It is hypothesized that (1) the soccer players (contact sport) will demonstrate greater postural stability compared to the baseball players (limited contact) and controls, especially when vision is removed, and that (2) this effect will become more pronounced as the difficulty of the task increases. More generally it is hypothesized that (3) less postural stability will demonstrated as the supporting area of the task decreases, especially in eyes-closed condition, however, as hypothesized above this effect will be less pronounced in the contact sport group.

## Methods

### Subjects

Thirty male college students, consisting of 10 collegiate soccer players (age = 21.5±1.9, height, 171.7±2.2cm; body mass, 64.3±4.8kg), 10 collegiate baseball players (age = 19.3±1.6, height, 174.3±4.0cm; body mass, 71.83±7.4kg), and 10 male students who had no special experience of any sport (age = 22.4±1.5, height, 173.3±3.6cm; body mass, 68.83±5.8kg) were recruited. The soccer and baseball players were selected based on a minimum of 8 years competitive training, playing only one sport, and having representation at primary, middle and high school, and university in Japan. The controls were selected based on not having taken part in any competitive sport or training. None of the participants had injuries inhibiting maximal exertion or conditions likely to be aggravated by maximal exertion. All participants agreed to the experimental procedure of the study that was specifically approved by the Human Research Ethics Committee in Faculty of Sport Sciences, Waseda University.

### Data collection

The participants were initially asked to stand barefoot in front of the force plate. When recording was initiated, the participants were instructed to step onto the force plate and adopt a randomly assigned posture. Once quiet balance had been achieved a trigger (including a tone) was activated. For each trial 60 seconds of data were recorded from the force plate (AMTI model OR6-5-1) which were sampled at 100 Hz. The data from the first 10 seconds after the trigger were chosen for analysis. Four tasks/postures of increasing difficulty were tested. In the first posture (bipedal stance), participants stood comfortably on both feet, separated as they desired. In the second posture (unipedal stance), participants stood on their customary supporting foot (e.g. the supporting leg when kicking a ball) while the other foot was lifted with the big toe placed alongside the medial malleolus of the supporting leg. In the third posture (unipedal_foam stance), participants stood on a 9 cm thick foam mat (16g/cm^3^) placed on top of the force plate in unipedal stance as described in the second posture. In the fourth posture (toe stance), the participants stood on their customary supporting foot and raised the heel, the other foot was lifted placing the big toe alongside the medial malleolus of the supporting leg.

The participants conducted each task with conditions of eyes-open and eyes-closed. When the participants had their eyes open, they were asked to fix their gaze on a letter ‘E’ (font size = 72) which was placed in front of them at eye level a distance of 5 m away. During the tests with eyes-closed, participants were asked to keep their “gaze” straight ahead [25] and maintain balance. In all trials, participants were instructed to keep their body straight with their hands on their hips. Participants performed three trials for each condition, so that 24 trials were completed for each participant. A one minute rest was taken between trials and the order of the 24 trials was randomized over the participants.

### Data processing

**Fig 1:**
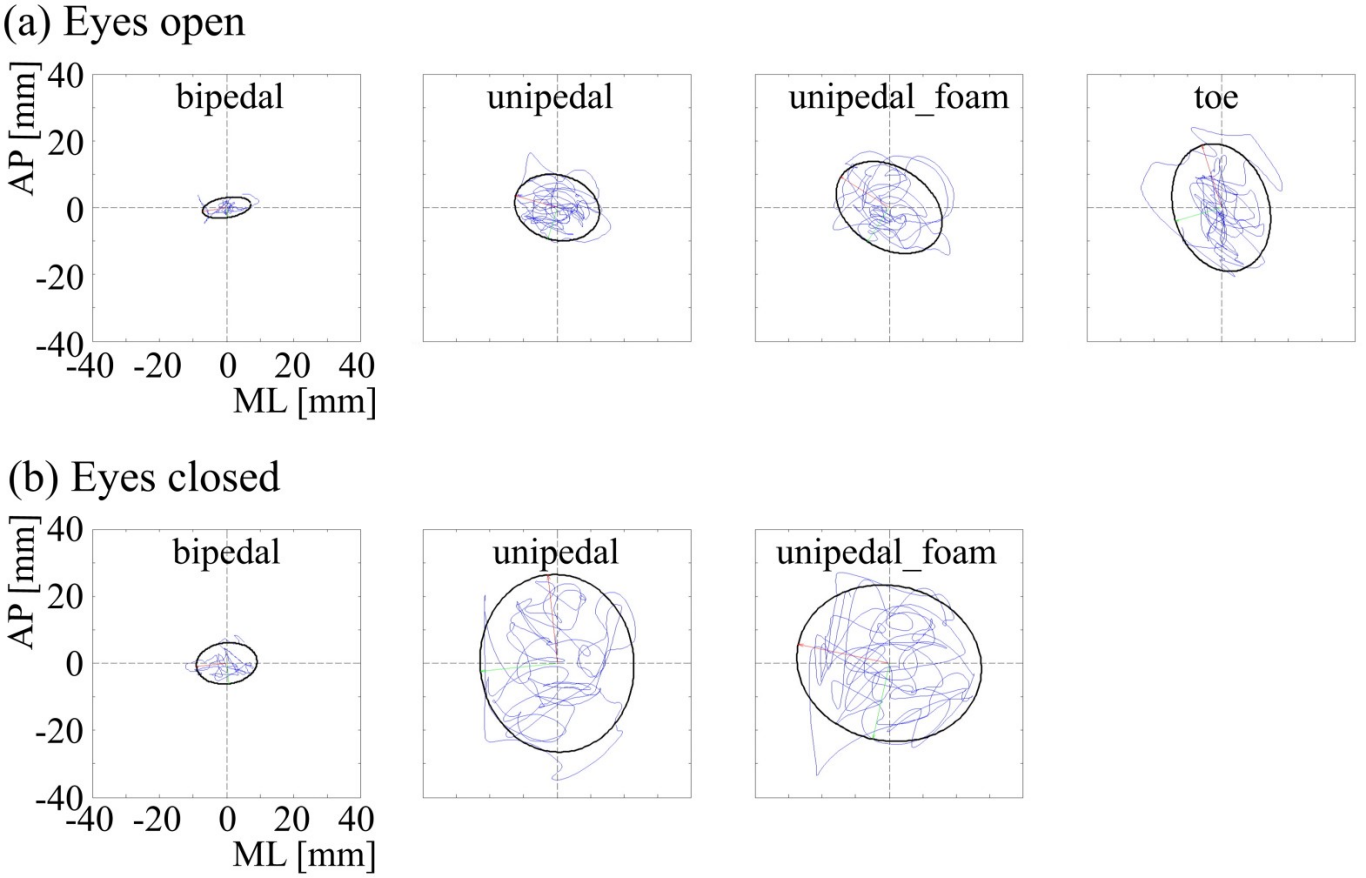
A typical example of the fluctuation of the COP in AP (anterior-posterior) and ML (medial-lateral) directions and the AOS as ellipse shown in a baseball subject with eyes-open and eyes-closed in the bipedal, unipedal, unipedal_foam, and toe stances.

The force plate data were low pass filtered with a second-order Butterworth filter (10 Hz). The displacement of the center of pressure (COP) in the anterio-posterior (AP) and medio-lateral (ML) directions was calculated from the vertical and horizontal reaction forces. Two dependent variables were used to investigate the participants’ postural behavior. The mean speed of the COP displacement (mm/s) was calculated by the sum of the displacement scalars (i.e. the cumulated distance over the sampling period) divided by the sampling time [25] using the following equation:

$$COP\;Speed=(\frac{1}{T})\sum_{i=1}^{N}\left|{COP}_{i}-{COP}_{i-1}\right|$$

where T is the time duration of the series and N is the total number of points in the series. The area of the stabilogram (AOS) was calculated by taking the ratio of the major and minor axes and then fitting an ellipse that included 85% of all the trajectory points [26] (Fig 1). COP speed and AOS were calculated using custom software written using MATLAB (The Mathworks Inc).

### Statistical analysis

Mean values for each dependent variable were calculated across the three trials for each condition and posture. Since all participants were unable to perform toe stance with eyes-closed, and only a small number of participants in the novice group were capable of performing toe stance with eyes-open, the main analysis excluded toe stance.

The effects of the group, conditions and tasks were evaluated, 3 (groups: soccer, baseball, and novices) × 2 (conditions: eyes-open and eyes-closed) × 3 (postures: bipedal, unipedal and unipedal with foam), using three-factor analysis of variance (ANOVA) for each dependent variable. The interaction between two factors was evaluated in the simple main effects. Post hoc tests were made using t-Tests with a Bonferroni correction. In addition one-way ANOVA of expertise in toe stance with eyes-open between groups was conducted to compare the effect of group on AOS and COP speed of postural sway. The Shapiro-Wilk test was used for normality, and homogeneity of variances was investigated using Levene’s test. Statistical significance was established a priori as p = 0.05 and partial eta squared (η^2^) was used to calculate the effect size (small = 0.01, medium = 0.06 and large = 0.14) [27].

## Results

**Fig 2:**
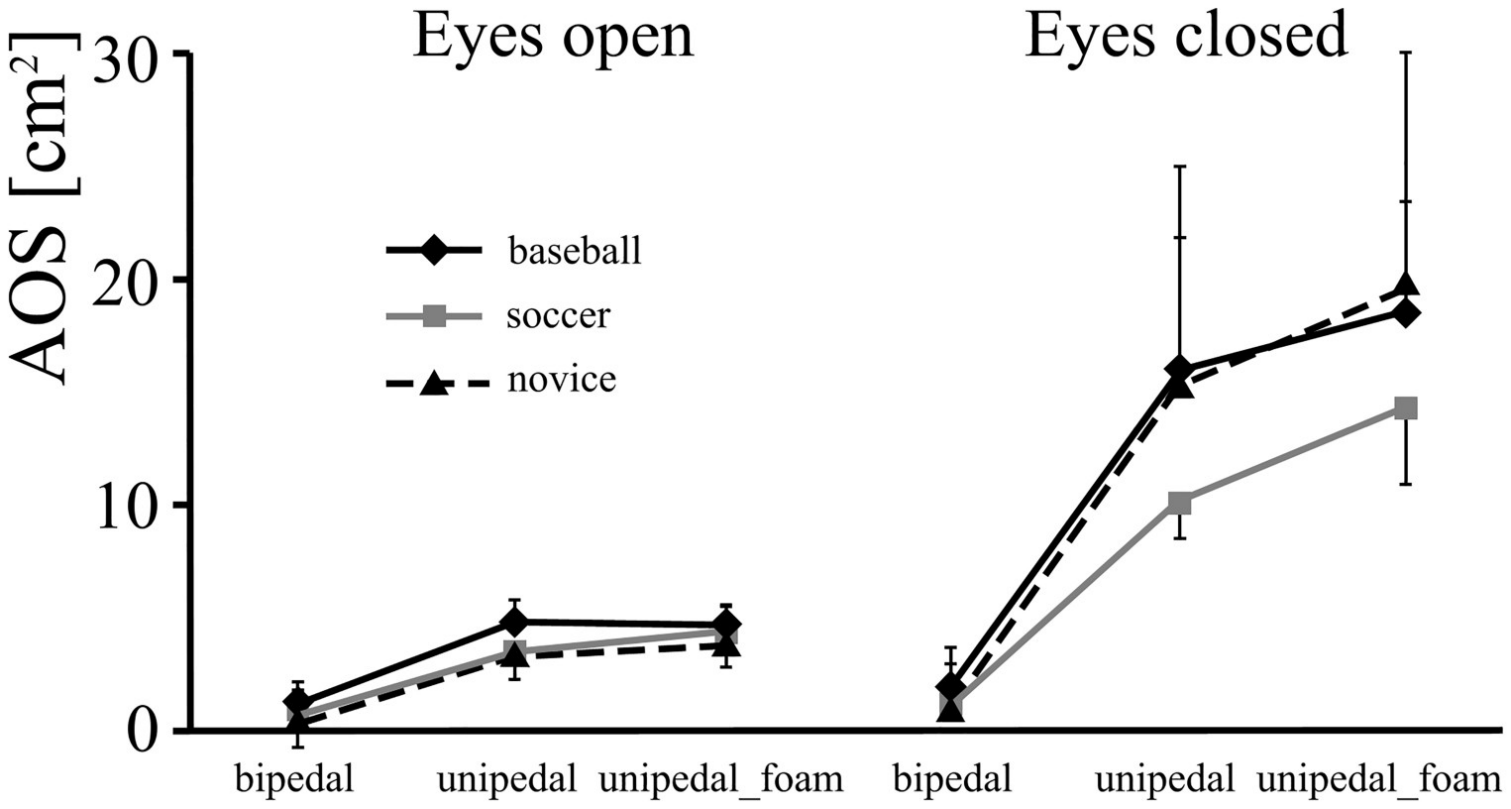
The AOS in the baseball, soccer and novice groups with eyes-open and eyes-closed in the bipedal stance, unipedal, and unipedal_foam stances.

The dependent variable of AOS revealed expertise (*F* (2,162) = 4.460, *p* < 0.05, η^2^ = 0.05), vision (*F* (1,162) = 174.458, *p* < 0.001, η^2^ = 0.52), and posture effects (*F* (2,162) = 103.480, *p* < 0.001, η^2^ = 0.56) and also significant two-way interactions of expertise and vision (*F* (2,162) = 3.714, *p* < 0.05, η^2^ = 0.04) (Fig 2), and of vision and posture (*F* (2,162) = 41.846, *p* < 0.001, η^2^ = 0.34). Soccer players had little sway in the eyes-closed condition in comparison to baseball players and novices for both unipedal and unipedal_foam. Postural sway in baseball players was comparable to that of the novices. Postural sway increased as the difficulty of posture increased only in the eyes-closed condition among the three groups.

**Fig 3:**
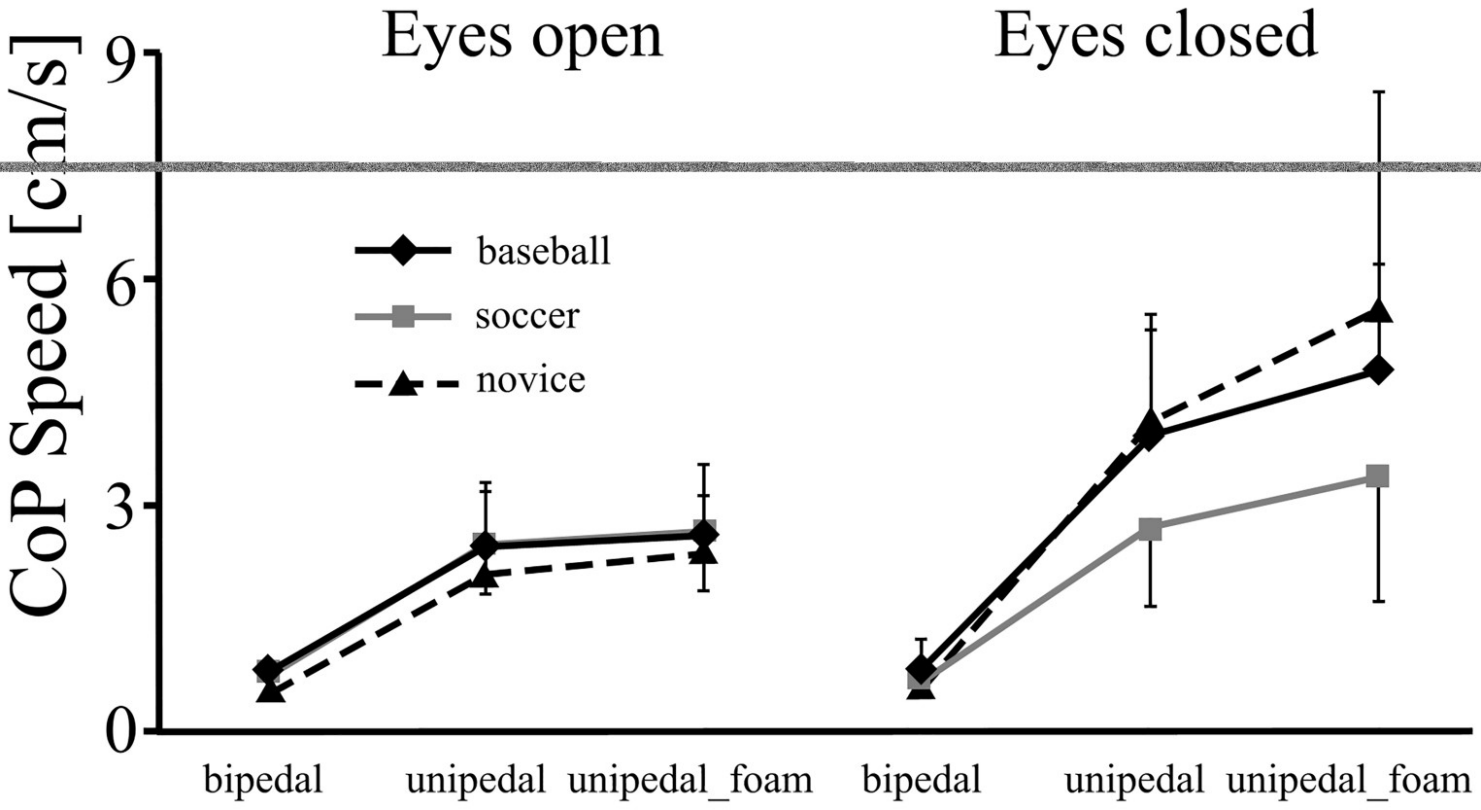
The COP speed in ML direction in the baseball, soccer and novice groups with eyes-open and eyes-closed in the bipedal, unipedal and unipedal_foam stances.

**Fig 4:**
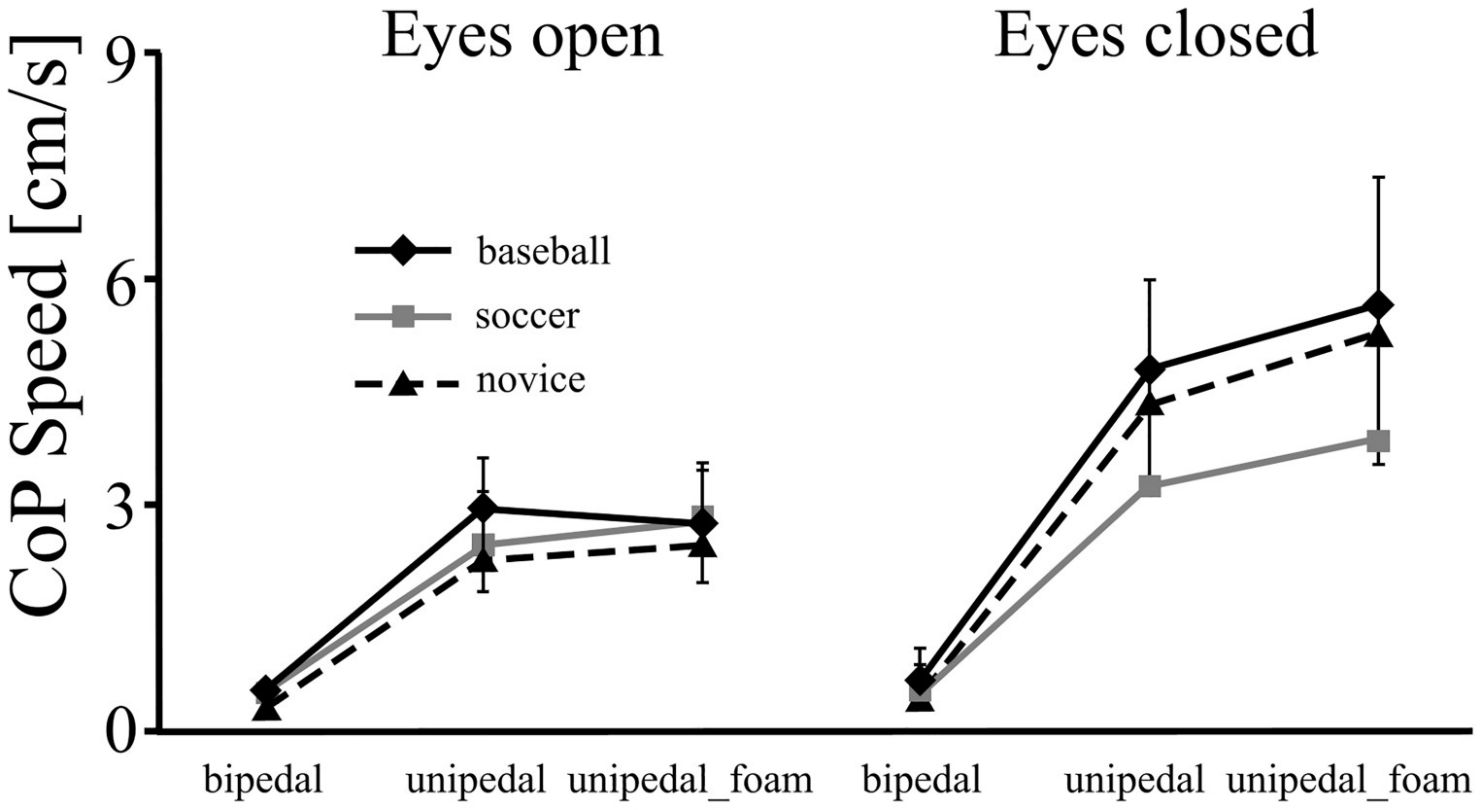
The COP speed in AP direction in the baseball, soccer and novice groups with eyes-open and eyes-closed in the bipedal, unipedal and unipedal_foam stances.

The dependent variable of COP speed in the ML direction and AP direction revealed vision ((*F* (1,162) = 131.597, p<0.001, η^2^ = 0.55) and (*F* (1,162) = 224.169, p<0.001, η^2^ = 0.58), respectively), and posture effects ((*F* (2,162) = 142.448, p<0.001, η^2^ = 0.64) and (*F* (2,162) = 308.479, p<0.001, η^2^ = 0.79), respectively) and also significant two-way interactions of vision and posture ((*F* (2,162) = 29.641, p<0.001, η^2^ = 0.27) and (*F* (2,162) = 53.235, p<0.001, η^2^ = 0.40), respectively)) (Figs 3 and 4). That is, the eyes-closed condition lead to the COP speed increasing more than in the eyes-opened condition only when the unipedal and unipedal_foam stances were performed.

The one-way ANOVA of expertise was made to clarify the effect of expertise with the amount of contact among three groups in the most challenging posture of toe stance. It showed there was no significant effect for expertise between the soccer, baseball and novice groups in regard to AOS of the toe stance in the eyes-open condition (*F* (2,24) = 0.451, *p* > 0.05, η^2^ = 0.04). There was also no significant difference in COP speed in the AP direction (*F* (2,24) = 1.670, *p* > 0.05, η^2^ = 0.10), and ML direction (*F* (2,24) = 1.255, *p* > 0.05, η^2^ = 0.12). Only seven out of ten participants in the novice group were able to perform the posture of toe stance while all the participants from the sports groups could.

## Discussion

Previous research has shown that expertise in sport results in superior postural control [14,18,19,28,29] although the effect of the amount of contact within those sports on postural control was still to be established. Additionally, across the range of available research the methods of assessing balance (field based and force plate) and the complexity of tasks (bipedal, unipedal, eyes open, eyes closed, on foam) has not been sufficiently consistent to make direct comparisons [14]. The purpose of the present study was to investigate whether an athlete’s participation in a contact sport such as soccer resulted in better postural control than those who participated in limited-contact sports such as baseball or those who did not participate in any sport. A significantly lower postural sway area was found for the soccer players (contact sport) compared with baseball players (limited contact) and novices (no contact), during uni-pedal stance and uni-pedal_foam stance under the condition of no vision. This result supports the hypothesis that expertise in contact sport has a positive impact on postural control. That the baseball group were comparable with the control group is in contrast to Davlin [30] who found that expertise in sport resulted in better dynamic balance than controls. It also confirms that more challenging tests of balance than previously used [12,13] are required to determine differences between the various levels of contact in different sports. However, comparable postural performance was found amongst all three groups during toe stance. In particular none of the participants was able to perform toe stance when vision was removed. This result only partially supports the hypothesis that as the task becomes more challenging, the benefits of expertise in contact sport become more pronounced in uni-pedal stances (Fig 3).

**Fig 5:**
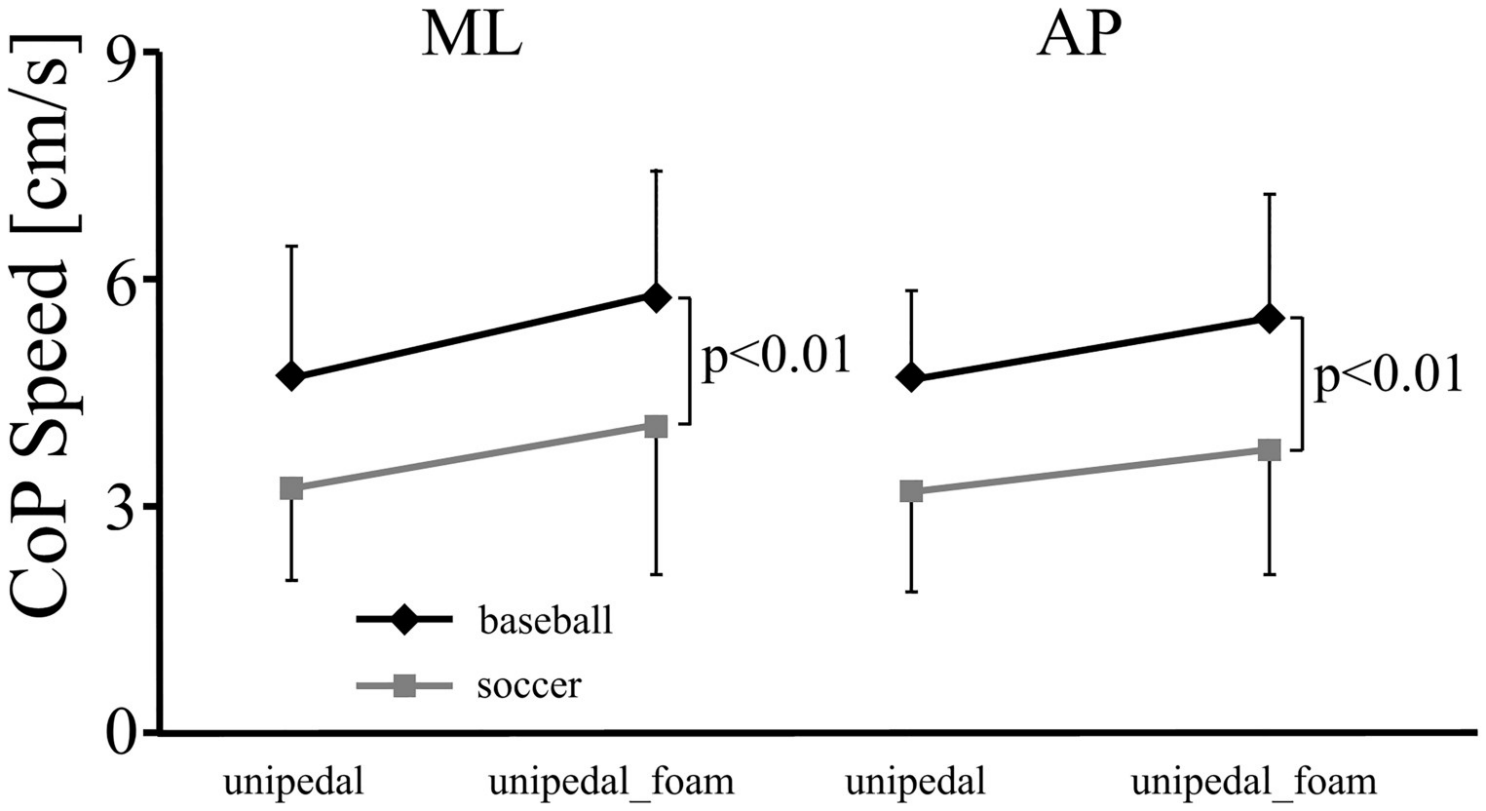
The COP speed in ML & AP directions in the soccer, baseball and novice groups with eyes-closed in the unipedal and unipedal_foam stances.

Increased postural stability may be developed with the diverse nature of expertise in sport, similar to being trained on an unstable compared with a stable surface [29] and requiring ‘dynamic balance’ as opposed to ‘static balance’ [19]. The aforementioned results can only show that contact experience benefits postural control in general. Whether the amount of contact experience would lead to differential effects on postural control, is still to be established. Thus, additional statistics (two-way ANOVA) were carried out in the distinguished stances of unipedal and unipedal_foam without vision, between just the soccer and baseball groups. Both AOS and COP speeds (ML and AP) in the soccer group were significantly lower than the baseball group (*F* (1,36) = 13.220, *p* < 0.01, η^2^ = 0.27; *F* (1,36 = 8.915, *p* < 0.01, η^2^ = 0.20; *F* (1,36) = 11.878, *p* < 0.01, η^2^ = 0.25) (Fig 5). This, together with above results, confirmed the hypothesis that the contact group demonstrated greater postural stability than the limited-contact and non-sport groups, with special attention on the eyes-closed condition in unipedal stances. These findings have implications for the study of postural control in sport, as the level of expertise and contact experienced by the participants will have an effect on postural control. That is, care should be taken to avoid any “contact sport bias” when selecting participant groups.

It has been suggested that for healthy people the sensory contributions to quiet standing are 70% from somatosensory, 20% from vestibular and 10% from visual information [31,32]. In the present study, postural performance became worse when visual information was removed (Fig 2). It was also observed that the soccer players had significantly less dependence on vision compared to the other participants, which would suggest that they were better able to use somatosensory and vestibular information when vision was removed. This result is similar to those shown in a study on gymnasts and dancers by Vuillerme et al. [16], who suggested that gymnasts are capable of using the remaining sensory information to keep posture stable even with the loss of vision. Golomer et al. [33] and Paillard and Noe [10] presumed that soccer players and ballet dancers were able shift the sensorimotor dominance from vision to proprioception. It is interesting that specific balance skills are often practiced in gymnastics and dance, which might be expected to result in more efficient utilization of vestibular and proprioception sensory information. However, soccer is not supposed to highlight any particular balance training, but players seem more able to transfer to proprioception and vestibular systems when vision is not available, compared to baseball players and the untrained participants. Bressel et al. [34] also reported that soccer players and gymnasts did not differ in balance tests. Presumably, physical contact training works well for improving postural control. Perrin et al. [12] found that judo players, a sport which would be defined as a collision sport, performed better than dancers in bipedal stance with the eyes closed. It could be argued that judo, unlike soccer, involves specific balance training since one of the goals of the sport is to avoid being toppled by an opponent. Additionally, study [12] did not say how sports with more limited contact would compare.

Previous research has shown that soccer players demonstrate superior balance compared to basketball players and controls [11,34,35,36]. Basketball could be classed in the contact sport group, however, given that excessive contact is penalized by the referee, and the evidence from previous studies, it would fall into the limited contact group alongside such sports as baseball and squash. Again, it is difficult to directly compare results due to the limited number of conditions used and the predominantly field based testing used. Although, the present experimental designed aimed to resolve this issue, there are still limitations with the present study. The present cohort of participants was drawn from collegiate athletes and was relatively low in numbers. However, collegiate athletes have been used extensively in the literature [14,28,34,35,37] and all participants had undergone extensive training in their one sport for numerous years. While it would have been ideal to have a larger sample size, the effect sizes found in the present study were meaningful [26], and based on a review of the area [14] the majority of studies comparing balance in a variety of sports have also had comparatively small sample sizes [2,10,11,13,14,16,34,35,37,38].

What remains unclear is whether, by having a challenging stance, effects on postural control could arise from changes in the area of the base of support, support surfaces, or both. Introducing less supporting area might thus reveal a more complex phenomenon amongst subjects. However, the soccer players were not more stable in toe stance in the eyes-open condition. There are two possibilities; one is that the task of toe standing is overwhelming for all participants, which is supported by the completely failed trials in toe-standing with eyes-closed in the present study. The other is that the participants were using the control strategy (eg. ankle and hip strategies) for bipedal stance in the toe stance condition. Nolan & Kerrigan [20] concluded that despite more open loop corrections, there were no significant differences in the closed loop control between toe standing and bipedal stance. The present finding of comparable postural performance amongst the three groups in bipedal stance may indirectly support the latter possibility (Figs 2, 3 and 4). This finding is also consistent with results from previous studies where specific training experience has been shown to have a small effect on fine postural control in bipedal stance [1,2]. Bipedal stance was therefore found to be limited in revealing differences in postural stability due to transfer from particular training [39,40,41]. This is likely due to the somatosensory stimulation being below the physiological threshold leading to an intermittent process [42,43,44]. This implies that when attempting to establish difference in postural control between players of various sports it is necessary to design tests that are suitably challenging.

It may be argued that having a smaller AOS and lower COP speeds are indicative of participants who possess steady posture control within a changed environment. Results from the additional analysis support this view, with significantly less postural sway in the contact sport group compared with the limited contact group under the non-vision condition. Biec & Kuczynski [17] proposed that soccer players exhibited different postural strategies from novices with a lower rate of postural corrections, more feedforward control and higher postural automaticity. As Deveau et al. [45] reported, specific training alters the brain so it is better able to respond to real life situations. More specifically, soccer players who were pushed off balance in such situations would react in a way that closely resembled balance training, particularly when the original balance was broken by an external disturbance. Thus, the proprioception and vestibular sensory systems are evoked and provide necessary input channels for sensory information when soccer players are working on ball control and combatting physical disturbance by an opponent. This would help explain why the soccer players are better able to cope with the loss of visual information. In that regard, contact-sport training such as in soccer may improve the proprioceptive and vestibular functions relevant for retaining balance, as sensory reweighting occurs when sensory systems change with environmental conditions [24,46].

It remains unclear whether the soccer players are better at detecting relevant sensory information or whether they are better able to respond to the information compared to the baseball players, due to having acquired different postural control strategies. According to Horak [32], there are two main types of movement strategies used to maintain balance during quiet stance, the ankle strategy and the hip strategy. It has been suggested that the former strategy is used for small perturbation in situations such as bipedal standing, and the latter strategy is used for larger perturbations, as in heel-toe standing [47]. In the same unperturbed stance while on a hard surface or with a foam support, Mesure et al. [48] confirmed that the experts with sport training preferred the ankle strategy, but the controls chose to use the hip strategy. Hence, the selection of postural strategy seems to be related to previous experience in sport, so that for a given situation the player is able to select the most appropriate strategy in order to respond to the perturbation [32,49,50].

## Conclusion

Participants involved in sport with physical contact (soccer) were found to have superior postural control compared with participants involved in sport with limited contact (baseball). This was particularly evident during the more challenging unipedal stance. Routine participation in sport involving physical contact appears to be an effective method for training proprioceptive and vestibular plasticity to posture control, particularly when vision is lacking.

## Supporting information

S1 File Sway area and sway speed data for all participants. (CSV)
